# Recent Findings on AMPA Receptor Recycling

**DOI:** 10.3389/fncel.2018.00286

**Published:** 2018-09-03

**Authors:** Edoardo Moretto, Maria Passafaro

**Affiliations:** Institute of Neuroscience, Consiglio Nazionale delle Ricerche (CNR), Milan, Italy

**Keywords:** AMPA-Rs recycling, LTP, LTD, recycling endosomes, homeostatic plasticity

## Abstract

α-Amino-3-hydroxy-5-methyl-4-isoxazolepropionic acid receptors (AMPA-Rs) are tetrameric protein complexes that mediate most of the fast-excitatory transmission in response to the neurotransmitter glutamate in neurons. The abundance of AMPA-Rs at the surface of excitatory synapses establishes the strength of the response to glutamate. It is thus evident that neurons need to tightly regulate this feature, particularly in the context of all synaptic plasticity events, which are considered the biological correlates of higher cognitive functions such as learning and memory. AMPA-R levels at the synapse are regulated by insertion of newly synthesized receptors, lateral diffusion on the plasma membrane and endosomal cycling. The latter is likely the most important especially for synaptic plasticity. This process starts with the endocytosis of the receptor from the cell surface and is followed by either degradation, if the receptor is directed to the lysosomal compartment, or reinsertion at the cell surface through a specialized endosomal compartment called recycling endosomes. Although the basic steps of this process have been discovered, the details and participation of additional regulatory proteins are still being discovered. In this review article, we describe the most recent findings shedding light on this crucial mechanism of synaptic regulation.

## Introduction

α-Amino-3-hydroxy-5-methyl-4-isoxazolepropionic acid receptors (AMPA-Rs) are tetrameric ionotropic receptors made up of preassembled dimers of four different, although highly homologous, subunits: GluA1-4. The most common dimers in the adult central nervous system of mammals are GluA1/2 and GluA2/3 (Huganir and Nicoll, 2013).

AMPA-Rs respond to the binding of the neurotransmitter glutamate by opening their central channel thus leading to the entry of sodium (and calcium if lacking the GluA2 subunit) and the exit of potassium ions allowing for depolarization of the postsynaptic neuron (Scannevin and Huganir, 2000; Henley and Wilkinson, 2016).

These receptors are crucial for basal excitatory transmission and are among the most important in synaptic plasticity phenomena, namely, Hebbian (long term potentiation, LTP or long term depression, LTD) or homeostatic plasticity, since their abundance at the postsynapse regulates the strength of the response to presynaptic release of glutamate (Henley and Wilkinson, 2016).

In particular, the increase of AMPA-Rs is typical, and necessary, for all of the forms of LTP and in the response to prolonged activity blockade. On the other hand, LTD and long-lasting activity enhancement lead to AMPA-R reduction at the postsynapse (Huganir and Nicoll, 2013).

Apart from the insertion of receptors newly synthesized (either distally or locally; Ju et al., 2004), two main pathways are exploited by neurons to regulate AMPA-R presence: lateral surface membrane diffusion from extrasynaptic sites (Tardin et al., 2003; Groc et al., 2004) and cycling between synaptic surface and intracellular endosomal compartments (Shi et al., 1999).

In this review article, we will focus our attention on the latter of the two processes since it is probably the one that has been most extensively described in the literature (for other reviews, see Hirling, 2009; Hanley, 2010; van der Sluijs and Hoogenraad, 2011; Henley and Wilkinson, 2013; Widagdo et al., 2017).

AMPA-Rs, similarly to many other surface proteins, are not only localized at the postsynaptic density but are also found in intracellular compartments such as in early endosomes, where they localize upon endocytosis, in late endosomes, where they are directed for degradation, and in recycling endosomes, specialized organelles that are able to translocate to the cell surface for delivery of transmembrane proteins (Scannevin and Huganir, 2000; Henley and Wilkinson, 2016). This system, namely, the endosomal system, is crucial for the regulation of surface proteins levels in almost every cell of the human body (Maxfield and McGraw, 2004).

This pathway leads to the existence of a continuous cycling of AMPA-Rs between these compartments and provides neurons with a pool of inactive intracellular receptors that are ready to be replenished or rapidly delivered upon stimuli such as synaptic plasticity (Hirling, 2009).

Mammalian cells present two pathways of recycling, one named “long loop” that involves the transport of endocytosed molecules to the pericentriolar endosomal system and one named “short loop” in which proteins are locally redirected back to the plasma membrane (Li and DiFiglia, 2012).

In the short loop, which in neurons can occur in close proximity to dendritic spines, endocytosed proteins are localized in a functional compartment named “sorting endosomes” in which their fate is decided. The pH of this compartment is acidic enough (pH ~ 6) to dissociate the majority of ligands from their receptors (Maxfield and McGraw, 2004). The sorting endosome is composed of large vacuoles, which could mature and fuse with the late endosomal compartment, and of tubular structures, which are thought to become part of the recycling endosomal compartment (Li and DiFiglia, 2012).

Although exclusive markers for recycling endosomes are lacking, different proteins have been shown to participate in the function of recycling endosomes and are usually used to identify this compartment.

The most important of these is likely the small GTPase Ras-related protein Rab11 (Ren et al., 1998). The function of Rab11 is mediated by different effectors that include Rab11 family interacting proteins (Rab11-FIPs; Hales et al., 2001) and the motor proteins MyosinVa/b, which are thought to be the transporter of recycling endosomes (Lapierre et al., 2001; Hales et al., 2002) and are also specifically involved in AMPA-Rs recycling (Correia et al., 2008). Rab8 and Rab35 GTPases have been found to participate in the exocytosis of recycling endosomes (Kouranti et al., 2006; Brown et al., 2007; Jullié et al., 2014).

Neuron-specific features of exocytosis of recycling endosomes have been discovered. In addition to common rapid exocytosis and release of membrane proteins to the plasma membrane, neurons present to a greater extent a second modality of exocytosis named persistent or display (Jullié et al., 2014). In this modality, the recycling endosomes are fused in a “kiss and run” fashion with opening and closure of a fusion pore. This mechanism leads to the retention of receptors in the membrane of recycling endosome and thus in restricted areas of the plasma membrane. This phenomenon is the most prevalent when observing recycling endosomes containing the Transferrin Receptor, AMPA-Rs and β2 adrenergic receptor (Jullié et al., 2014). Evidence also suggests that recycling endosomes are subdivided into different pools containing different receptors (i.e., AMPA-Rs and β2 adrenergic receptors; Jullié et al., 2014). Many adaptor proteins participate in regulating all the steps of AMPA-R cycling (for review see Hirling, 2009; Anggono and Huganir, 2012; Bassani et al., 2013).

**Figure 1:**
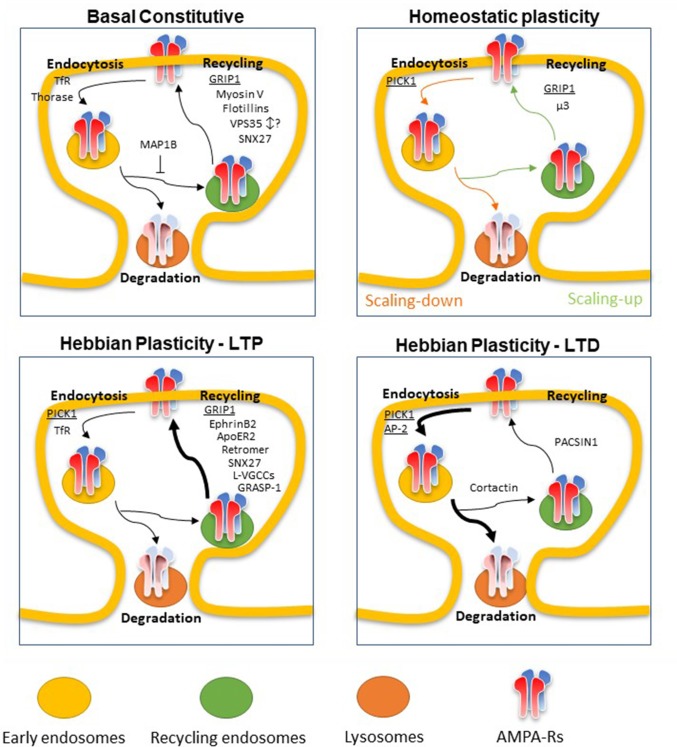
Scheme of newly discovered adaptors of α-Amino-3-hydroxy-5-methyl-4-isoxazolepropionic acid receptors (AMPA-Rs) recycling in Basal constitutive condition or upon Homeostatic or Hebbian (long term potentiation, LTP or long term depression, LTD) plasticity. Major proteins involved in AMPA-R recycling are underlined. Basal constitutive: AMPA-Rs are constitutively endocytosed and sorted between lysosomes for degradation or recycling endosomes for recycling back to the plasma membrane. Glutamate interacting protein 1 (GRIP1) is one of the main adaptors driving AMPA-R exocytosis by binding to the C-terminal tail of GluA2. Myosin V is the main actin motor involved in delivery of AMPA-Rs-containing recycling endosomes to the plasma membrane. Flotillins Reggie-1 and -2 promote the recycling of AMPA-Rs whereas TfR is mainly involved in its endocytosis in basal condition. Thorase was found to regulate AMPA-R endocytosis by disrupting the interaction between GRIP1 and AMPA-R subunit GluA2. The retromer complex, and more specifically vacuolar sorting proteins 35 (VPS35), and its adaptor SNX27 have been found to participate in AMPA-R recycling in basal condition. SNX27 was found to positively regulate the process whereas discording results have been found for VPS35. MAP1B was found to retain AMPA-Rs away from dendritic spines preventing the entry into the recycling endosomal system by interacting with GRIP1. Homeostatic plasticity: depending on whether the plasticity is a synaptic scaling-up (after chronic activity blockade, green arrows) or scaling-down (after chronic activity enhancement, orange arrows), AMPA-Rs are mainly internalized, with the help of PICK1 binding to GluA2 C-terminal tail and degraded or recycled back to the surface membrane thanks to GRIP1 action, respectively. μ3 subunit of the adaptor complex AP-3A promotes the recycling of AMPA-Rs to the plasma membrane in scaling-up phenomena. Hebbian plasticity-LTP: LTP induces an increase in synaptic abundance of AMPA-Rs mainly promoting the exocytosis of an internal pool of receptors. GRIP1 is one of the main adaptor proteins exerting this action whereas PICK1 has been found to induce GluA2-containing AMPA-R endocytosis also upon LTP stimuli, possibly to allow the temporary substitution with CP-AMPA-Rs. The retromer complex and its adaptor SNX27 have a positive effect on AMPA-R exocytosis during LTP. GRIP associated protein 1 (GRASP1), ApoER2 and ephrinB2 also promote AMPA-R surface delivery, through the interaction with GRIP1. L-VGCCs have been shown to have a crucial role, via the increase of intracellular calcium, in causing a complete fusion of recycling endosomes to the plasma membrane upon LTP stimuli. Hebbian plasticity-LTD: Long term depression is induced and maintained by an increase in internalization rates of AMPA-Rs followed by lysosomal degradation. PICK1 is crucial in AMPA-R removal from the surface membrane through its interaction with GluA2 and with the adaptor protein complex AP-2. Cortactin association with AMPA-Rs was found to counteract this process promoting the sorting of the receptors toward recycling endosomes. PACSIN1 was involved in promoting recycling endosomes exocytosis thus acting against the induction and maintenance of LTD.

Our review article will focus on the most recent findings obtained both on the definition of the mechanism of AMPA-R recycling and on newly discovered adaptor proteins in basal constitutive recycling and recycling in synaptic plasticity (Figure 1).

## Basal Constitutive Recycling

Basal recycling is the cycling of AMPA-Rs between the plasma membrane and the endosomal compartment that occurs under basal conditions, independently from synaptic plasticity. AMPA-Rs are believed to undergo endocytosis primarily through clathrin and dynamin (Carroll et al., 1999; Man et al., 2000; Anggono and Huganir, 2012), although a clathrin-dynamin-independent endocytosis mechanism relying on actin dynamics has been observed (Glebov et al., 2015). Endocytosis is thought to occur in the Endocytic Zone (EZ), a region localized just outside the postsynaptic density (Lu et al., 2007).

Once internalized, the receptors might be relocated to recycling endosomes for delivery back to the plasma membrane or to the lysosomal compartment for degradation.

While this is a general process of AMPA-R basal recycling, it is important to note that there are AMPA-R subunit specific mechanisms which will be discussed later in “Basal Constitutive Recycling” section.

The next paragraph will focus on the study of newly discovered adaptor proteins that regulate AMPA-R recycling.

Recent work has elucidated part of the basal motor proteins involved in the delivery of AMPA-R-containing recycling endosomes to the plasma membrane (Esteves da Silva et al., 2015). Esteves da Silva et al. (2015) have taken advantage of the recently developed chemically inducible dimerization system FRB-FKBP (Kapitein et al., 2010) to induce binding between motor proteins and Rab11-positive recycling endosomes that contain AMPA-Rs. This study showed that the microtubule motor KIF1C and the actin motor Myosin V are both involved in this process in accordance with previous findings (Setou et al., 2002; Wang et al., 2008). The motor myosin VI was instead found to participate in the removal of Rab11-positive recycling endosomes away from the synapse. In agreement with the well-known role of AMPA-Rs in regulating the strength of excitatory synapses, forcing the removal of Rab11-positive recycling endosomes from dendritic spines led to a general reduction of synapse strength both functionally and structurally.

In recent years, most of the studies have focused on the investigation of adaptors or regulatory proteins acting on AMPA-R recycling.

Among recycling regulatory proteins whose actions affect AMPA-Rs trafficking nonspecifically, the Rab11A binding proteins Reggie-1 and -2 were recently investigated in cultured hippocampal neurons and in a knockout mouse model (Bodrikov et al., 2017). These proteins, also known as flotillins, reside in lipid rafts and were previously found to bind Rab11A and SNX4, exerting crucial roles in the recycling of Transferrin Receptor, E-Cadherin, α5 and β1 integrins and T-Cell receptor in various cell lines (Stuermer et al., 2004; Stuermer, 2010; Solis et al., 2013; Hülsbusch et al., 2015) and in the sorting of N-Cadherin to the growth cone in neurons (Bodrikov et al., 2011). The exact mechanism of action of flotillins on Rab11A and/or SNX4 activities remains to be determined.

The authors discovered that the absence of Reggie proteins was linked with impaired recycling of AMPA-R subunit GluA1 together with *N*-methyl D-Aspartate (NMDA) receptor subunit GluN1 and N-cadherin under basal condition in neurons. A reduction of PSD-95 was also observed, leading to the hypothesis that Reggie proteins are general players in Rab11A-mediated recycling of synaptic proteins. This was also corroborated by the observation that the defects could be reversed by the overexpression of a constitutively active Rab11A (Bodrikov et al., 2017).

A recent study surprisingly discovered a regulatory effect of the TfR protein in the cycling of AMPA-Rs in neurons (Liu et al., 2016).

TfR, which binds to diferric transferrin and is crucial for iron homeostasis in the body, is highly expressed in neurons (Moos, 1996) and undergoes a high rate of cycling between the cell surface and the endosomal compartment in basal condition (West et al., 1997). For this reason, TfR has often been used as a control protein for studies of endocytosis and recycling of synaptic proteins, including AMPA-Rs. In this recent work, the absence of TfR appeared to cause a decrease in the association between AMPA-R subunit GluA2 with the endocytosis adaptor AP-2, thus slowing AMPA-R endocytosis. On the other hand, the recycling of AMPA-Rs occurred at faster rates with the net effect of higher levels of both GluA1 and GluA2 on the surface membrane. However, the authors propose that TfR acts indirectly on AMPA-Rs through regulation of the interaction of the latter with AP-2 (Liu et al., 2016). This possible competition mechanism remains to be directly demonstrated, and the possibility of more general unspecific defects of the recycling machinery upon TfR knockout needs to be ruled out.

The retromer complex is another group of proteins that have been shown to regulate the recycling machinery (Bonifacino and Hurley, 2008) and recent work has elucidated its effect on AMPA-R trafficking.

This complex, made up of the assembly of vacuolar sorting proteins 35 (VPS35), VPS26, VPS29 and different adaptor proteins, acts by sorting transmembrane protein away from lysosomal degradation for recycling from the endosomal compartment to the trans-Golgi network or to the plasma membrane (Bonifacino and Hurley, 2008). AMPA-Rs have been identified as one of the cargo of the retromer complex as demonstrated by the decrease in AMPA-evoked currents upon knockdown of VPS35 (Choy et al., 2014).

Different studies have linked the retromer complex to neurological and neurodegenerative conditions (Small and Petsko, 2015). Recently, the p.D620N mutation in VPS35 has been found to be associated with Parkinson’s disease (Munsie et al., 2015). This mutant protein showed a loss of function effect lacking the activity of wild-type VPS35 in decreasing AMPA-mediated transmission both in mouse cortical neurons and dopaminergic neurons derived from human patients. VPS35 was shown to interact with GluA1, to a greater extent compared with GluA2, suggesting subunit specificity of the complex. This selective action on GluA1 also supports a more prominent role of the retromer complex in activity-dependent recycling of AMPA-Rs.

Apparently discording results have been observed in a mouse model in which VPS35 was in heterozygosity. Decreased levels of VPS35 were associated with impaired AMPA-Rs trafficking to the cell surface, with a more relevant effect on GluA1 and a consequent defect in dendritic spines density and maturation in the CA1 region of the hippocampus. Interestingly, these effects on dendritic spines were reverted by GluA2 overexpression (Tian et al., 2015).

The similarity of effects on AMPA-Rs upon opposite modulation of VPS35 levels suggests that the exact stoichiometry of VPS35 is crucial for an efficient action on AMPA-Rs recycling. Additional effort is needed in order to clarify this aspect.

Sorting nexin 27 has been identified as one of the adaptor proteins that regulate cargo binding of the retromer complex (Temkin et al., 2011). As other members of the sorting nexin family, it presents a phox-homology domain (PX) that binds phosphatidylinositol phosphate (PIP; Teasdale and Collins, 2012) and can thus interact with either endosomes or the plasma membrane. SNX27 also presents a peculiar PDZ domain through which it can interact with GluA2 and GluA1 C-termini (Hussain et al., 2014). In addition, mutations or deficits in SNX27 have been associated with different neurological conditions in which impaired AMPA-mediated transmission plays a critical role (Wang et al., 2013; Damseh et al., 2015; Zhang et al., 2018). SNX27 positive regulation of AMPA-Rs exocytosis in cultured hippocampal neurons was assessed by surface staining upon either overexpression or knockdown of SNX27 and a role in this process was established for both the PX and PDZ domains (Hussain et al., 2014).

Glutamate interacting protein 1 (GRIP1) is believed to be one of the major adaptor proteins regulating the fate of AMPA-Rs during all steps of intracellular trafficking thanks to the binding of the C-termini of the receptor through its PDZ domains (Dong et al., 1997; Anggono and Huganir, 2012). GRIP1 was shown to also interact directly with the kinesin motors (Setou et al., 2002) or in a complex with liprin-α (Wyszynski et al., 2002), promoting AMPA-R transport to dendrites and dendritic spines. In addition, its interaction with NEEP21 has been involved in AMPA-R sorting and more specifically recycling (Alberi et al., 2005; Steiner et al., 2005). Although the major role of GRIP1 in promoting AMPA-Rs exocytosis is generally accepted, other contrasting effects have been observed when interfering with GRIP1 function with apparent regulation of endocytosis and intracellular retention (Daw et al., 2000; Lu and Ziff, 2005). The picture is complicated by the fact that the GRIP1 binding site on the GluA2 C-terminus is shared with PICK1, a protein well known for negatively regulating AMPA-R surface levels by enhancing internalization and retention. The selectivity of the binding is regulated by the phosphorylation status of Serine 880 and of Tyrosine 876 of GluA2 C-terminal tail (Matsuda et al., 1999; Chung et al., 2000; Fu et al., 2003; Hayashi and Huganir, 2004).

Recent work has elucidated another level of complexity of this sophisticated machinery. GRIP1 has been found to present the ability to bind simultaneously both AMPA-Rs and N-Cadherin and associating them with the KIF5 motor protein, thus promoting their contemporaneous dendritic delivery, which would provide synapses instantly with a more complete machinery for establishment and maturation (Heisler et al., 2014). Other work has elucidated the already described (Seog, 2004) unusual interaction between GRIP1 and the MAP1B light chain in regulating AMPA-R trafficking under basal conditions (Palenzuela et al., 2017). MAP1B decorates microtubules along the dendrites (Halpain and Dehmelt, 2006) and is directly involved in dendritic spines morphogenesis (Tortosa et al., 2011). MAP1B light chain overexpression was shown to impair AMPA-mediated currents due to a specific reduction in the surface levels of the GluA2 subunit of AMPA-Rs. This effect, which was not associated with any defects in LTP, was likely caused by a reduction in dendritic targeting of GRIP1 that is proposed to be trapped by MAP1B outside the dendritic spine. This mechanism describes an unusual role for GRIP1in reducing synaptic delivery of AMPA-Rs. However, further investigation is needed, especially because a MAP1B mutant lacking the microtubule binding domain was shown to retain the ability to impair AMPA-mediated currents, arguing against the binding of MAP1B to microtubules as the trapping mechanism for GRIP1-GluA2 complexes (Palenzuela et al., 2017).

AMPA-R association with GRIP1 has recently been found to also be regulated by the protein Thorase, an AAA+ ATPase encoded by the ATAD1 gene (Zhang et al., 2011). This mechanism is of particular interest considering that different mutations in the ATAD1 gene have been found in patients affected by lethal encephalopathy (Ahrens-Nicklas et al., 2017; Piard et al., 2018). Thorase was proposed to become part of the complex between GRIP1 and GluA2 to disrupt their interaction after ATP hydrolysis and promote AMPA-R endocytosis (Zhang et al., 2011).

Accordingly, thorase knockdown or knockout, and encephalopathy-associated mutations, were found to impair AMPA-R internalization, causing increased levels of the receptor on the surface membrane, thus leading to the exaggerated excitatory transmission typical of epilepsy (Zhang et al., 2011).

One of the most elusive aspects of AMPA-R trafficking is the specificity of the various mechanisms and adaptors identified for different AMPA-R subunits.

As stated above, the most abundant AMPA-R in the adult mammalian central nervous system is made up of GluA1/GluA2 and GluA2/GluA3 dimers (Wenthold et al., 1996; Lu et al., 2009; Huganir and Nicoll, 2013) with a minor presence of GluA1/GluA1 homomers and GluA1/GluA3 heteromers at the postsynapse in basal condition. The presence of GluA2 in most of the receptors leads to technical difficulties in identifying specific endogenous mechanisms. In addition, there has been lower interest in elucidating properties of the GluA3 subunit. Previous studies have investigated the differential properties of GluA1 and GluA2 in trafficking and function (Passafaro et al., 2001; Shi et al., 2001).

However, most of the studies published do not address the complete subunit composition of the tetrameric receptors being analyzed.

As a whole, the literature mainly suggests that GluA2/GluA3-containing AMPA-R undergo rapid constitutive cycling between the synapse surface and the endosomal compartment, whereas GluA1-containing receptors are slowly recycled in basal conditions and are more effectively transported upon stimulation such as LTP (Passafaro et al., 2001; Shi et al., 2001).

Different studies have suggested the existence of another dimer: the GluA1/GluA1 homomer (Plant et al., 2006; Jaafari et al., 2012). The absence of GluA2 in this receptor allows it to become permeable to calcium ions and classified GluA1 homomers as Calcium-permeable AMPA-Rs (CP-AMPA-Rs) in contrast to GluA2-containing Calcium-impermeable AMPA-Rs (CI-AMPA-Rs). Current models predict that CP-AMPA-Rs are rarely present at the synapse under basal conditions in the adult brain, whereas they are rapidly delivered in the first moments of potentiating synaptic plasticity phenomena and then quickly reinternalized and substituted by CI-AMPA-Rs (Hanley, 2014). This implies that CP-AMPA-Rs are already assembled and rapidly released from an intracellular compartment, possibly the recycling endosomes. Variations in CP-AMPA-R levels have been seen in brain development and found to be associated with different pathological conditions including neuronal ischemia and cocaine addiction (Jaafari et al., 2012; Yuan and Bellone, 2013; Hanley, 2014). However, the precise mechanisms regulating CP-AMPA-Rs trafficking still need further clarification since most of the identified adaptor proteins are GluA2-specific interactors or binds indistinctly both GluA2 and GluA1.

A recent study addressed this topic in medium spiny neurons of the nucleus accumbens in mice (Werner et al., 2017). In these neurons, the accumulation of CP-AMPARs was demonstrated as a response to prolonged withdrawal after cocaine administration (Conrad et al., 2008). Werner et al. (2017) discovered that CP-AMPARs undergo faster endocytosis and recycling compared with CI-AMPARs in this paradigm.

However, further studies are needed to elucidate the specific properties of different AMPA-R tetramers to gain more precise knowledge of the behavior of these proteins.

## Synaptic Plasticity

### Homeostatic Plasticity

Homeostatic plasticity, also referred to as synaptic scaling, is a phenomenon of synaptic potentiation or depression that occurs upon a long-lasting decrease or increase of synaptic responses that can be reproduced *in vitro* by chronic administration of Tetradotoxin (TTX) or Bicuculline, respectively (Turrigiano, 2008).

Synaptic potentiation or depression occur through the up- or downregulation, respectively, in the abundance of AMPA-Rs at the plasma membrane, and this synaptic scaling is strongly regulated by endocytosis or recycling of AMPA-Rs (Wierenga et al., 2005; Gainey et al., 2009).

These plasticity phenomena are mainly believed to be adaptation responses to chronic, non-physiological stimuli, to restore a normal circuit signaling.

The opposing roles of GRIP1 and PICK1 strongly participate in regulating AMPA-R-levels under basal condition (see “Basal Constitutive Recycling” section above) and in Hebbian plasticity phenomena through their interaction with the GluA2 C-terminal tail (see “Basal Constitutive Recycling” section). More recently, these proteins have been involved in homeostatic plasticity with similar antithetical effects. PICK1 was shown to be crucial in homeostatic downscaling as these phenomena appeared occluded in cultured neurons from PICK1 knockout animals (Anggono et al., 2011). On the other hand, GRIP1 binding to GluA2 was enhanced in synaptic upscaling (Gainey et al., 2015; Tan et al., 2015).

A recent study on CP- and CI-AMPA-Rs cited above (Werner et al., 2017) also investigated the differential contribution of these receptors in synaptic scaling phenomena. Both long-lasting activity blockade and enhancement appeared to affect CI-AMPA-Rs to a greater extent compared with CP-AMPA-Rs, inducing increased recycling and exocytosis in the scaling-up plasticity and increased endocytosis in scaling-down phenomena.

Another interesting study pointed out the importance of the μ subunit of AP-3 complex in regulating AMPA-R recycling in mice after sensory deprivation, an *in vivo* correlate of synaptic scaling-up (Steinmetz et al., 2016). AP-3 belongs to a family of adaptor proteins, the adaptor protein complexes (APC), that are well-known regulator of endosomal trafficking by acting as vesicle coats (Bonifacino, 2014; Guardia et al., 2018). There are five known adaptor protein complexes (AP-1, -2, -3, -4, -5), all of them composed of four different subunits (Bonifacino, 2014; Guardia et al., 2018). AP-2, AP-3 and AP-4 have been shown to affect AMPA-R transport in the endolysosomal system (Burbea et al., 2002; Lee et al., 2002; Margeta et al., 2009; Matsuda et al., 2013). AP-3A was previously found to be involved in directing AMPA-Rs towards degradation in the lysosomal compartment upon LTD stimulation through the interaction with the transmembrane AMPA-R regulatory protein (TARP) stargazin (Matsuda et al., 2013). Steinmetz et al. (2016) through a transcriptomic analysis in pyramidal neurons of Layer 4 of the visual cortex after sensory deprivation, found that the transcription of the μ subunit of AP-3A was increased. In contrast to our knowledge of the adaptor protein complex family where the subunits are believed to be obligated tetramers, the μ3 subunit appears to act independently from the complex to recruit AMPA-Rs to the recycling endosomes.

### Hebbian Plasticity

Hebbian plasticity is considered the biological correlates of learning and memory. It refers to the ability of a pattern of stimuli with precise frequency and intensity to elicit the potentiation (LTP) or depotentiation (LTD) of synapses, reinforcing or weakening specific circuit connections. Both LTP and LTD rely on AMPA-R trafficking to modify the synaptic strength with increased surface delivery or endocytosis and degradation, respectively.

### Hebbian Plasticity—LTP

Different forms of LTP exist physiologically with the main one being dependent on NMDA-Rs. NMDA-R activation causes the increase of intracellular calcium, which activates a series of signaling cascades leading to the insertion of more AMPA-Rs at the postsynapse (Nicoll et al., 1988; Huganir and Nicoll, 2013). These insertion events take place through different mechanisms including increased exocytosis of recycling endosomes (Park et al., 2004; Huganir and Nicoll, 2013).

Recently, a new LTP mechanism relying on the activation of a metabotropic activity of kainate receptors to induce increased surface delivery of AMPA-Rs was identified (Petrovic et al., 2017). Kainate receptors are glutamate receptors that act in concomitance with AMPA-Rs in producing the depolarization of the postsynaptic neuron (Carta et al., 2014). The amplitude of their responses is generally lower compared with that of AMPA-Rs. Surprisingly, the authors identified a new mechanism of LTP induction based on the kainate receptor-mediated action of a G protein, not yet identified, that induces a signaling cascade of activation of protein Kinase C and Phospholipase C with the concluding effect of liberating recycling endosomes-containing AMPA-Rs and thus potentiating the responses to glutamate.

A very interesting study further investigated the molecular mechanism underlying these phenomena in chemical-LTP stimulated cultured neurons (Hiester et al., 2017). This work highlighted the need for a secondary calcium release through L-type voltage gated calcium channels (L-VGCCs) for the complete fusion of AMPA-R-containing recycling endosomes to the plasma membrane preventing its resealing without content release. Activation of NMDA-Rs without the subsequent activation of L-VGCCs, as with the application of specific inhibitors nimodipine, verapamil and diltiazem, appears to provide only the initial fusion of the vesicles with the membrane thus impairing the instauration of potentiation phenomena. Interestingly, the authors also showed, through high-resolution imaging experiments, that each synapse can contain multiple TfR-positive recycling endosomes.

The retromer complex, already introduced in the “Basal Constitutive Recycling” section, has been shown to also play a role in LTP-induced AMPA-R delivery. Very interestingly, depletion of the VPS35 subunit of the retromer complex *in vivo* in adult mice through lentiviral delivery of Sh-RNA caused the block of either NMDA-Rs- or L-Type Ca^2+^ channel-dependent LTP phenomena without affecting LTD (Temkin et al., 2017). These results are in agreement with the role of the retromer in AMPA-R delivery to the plasma membrane. However, in contrast to what described in the previous section, Temkin et al. (2017) did not observe any defect in basal AMPA-R-mediated transmission or in homeostatic plasticity-like treatment with retinoic acid. These findings argue against a general role for the retromer complex in all AMPA-R exocytosis phenomena as previously reported (Choy et al., 2014) and suggest on the other hand an LTP-specific involvement. Differences in model used possibly explain these inconsistencies; however, further research is needed to clarify these aspects.

Furthermore, the retromer adaptor SNX27 has recently been involved in LTP-driven AMPA-R delivery (Hussain et al., 2014; Loo et al., 2014). The work from Hussain et al. (2014) showed that the knockdown of SNX27 in cultured rat cortical neurons was sufficient to abolish the increase in surface-exposed GluA1 upon glycine treatment-induced chemical-LTP (Hussain et al., 2014). Loo and coworkers investigated a possible mechanism by which SNX27 exert its action on AMPA-R delivery. They observed that, shortly after glycine treatment, the membrane bound GTPase K-Ras, which is bound by Ca^2+^-activated Calmodulin (Villalonga et al., 2001; Wu et al., 2011), increases its interaction with SNX27. Concomitantly, the same stimulus enhanced SNX27 interaction with the GluA1 AMPA-R subunit and its surface delivery. The authors suggest a direct link between all these proteins in connecting the increase in Ca^2+^ concentration that follows LTP stimuli to increased GluA1 surface; however, direct evidences for the existence of this multimeric complex is needed.

TfR activity on AMPA-R recycling during LTP phenomena was evaluated in the study cited in the “Basal Constitutive Recycling” section (Liu et al., 2016). The authors took advantage of super-ecliptic pHluorins to study internalization and recycling of GluA1 and GluA2 subunits of AMPA-Rs in TfR knockout mouse-derived neurons upon brief NMDA application, a treatment that is known to trigger AMPA-R internalization. Both subunits showed impairment in endocytosis and faster recycling, which is in accordance with the observed increase in the surface level of the receptor.

However, quite surprisingly, LTP was reduced in intensity in CA1 synapses of TfR knockout mice. This might be an effect of LTP occlusion with the levels of AMPA-Rs being too high to be further enhanced, although this would have generated an increased input-output relation that was, instead, decreased. Further investigations are needed to clarify this aspect.

A specific function in LTP for GRIP1-mediated AMPA-Rs insertion, already introduced in the previous chapters, involving the interaction between ephrinB2 and ApoER2 has recently been described (Pfennig et al., 2017). ApoER2 act as a receptor for the secreted extracellular matrix protein Reelin to exert its action in promoting neuron maturation and positioning during migration through Dab1 (D’Arcangelo et al., 1999; Trotter et al., 2013). EphrinB2 was shown to participate in this Reelin-dependent mechanism (Sentürk et al., 2011). This pathway was also involved in hippocampal synaptic plasticity through the recruitment of GRIP1 by ephrinB2 (Essmann et al., 2008). In this work, Pfennig et al. (2017) showed that the multimeric complex between Serine-9 phosphorylated ephrinB2, ApoER2, GRIP1 and GluA2 was recruited under the condition of enhanced neuronal activity following KCl treatment of cultured neurons. Interestingly, in mice, reduction of levels of either ApoER2 or GRIP1 and expression of a mutant ephrinB2 deficient for Serine 9 phosphorylation all caused slight impairments in LTP induction and maintenance, whereas the simultaneous presence of all these modulations greatly enhanced the LTP defects (Pfennig et al., 2017).

Another very interesting article was published on the role of GRIP associated protein 1 (GRASP1) in AMPA-R recycling and LTP (Chiu et al., 2017). GRASP1 is a neuronal-specific Ras-GEF that interacts with GRIP1 and AMPA-Rs (Ye et al., 2000), which has been proposed to promote the transition from Rab4-positive early endosomes to Rab11-positive recycling endosomes (Hoogenraad and van der Sluijs, 2010; Hoogenraad et al., 2010). In this work, Chiu et al. (2017) characterized a GRASP1 knockout mouse identifying the crucial role of the GRASP1, GRIP1 and AMPA-Rs association in allowing correct trafficking of the receptor. Animals deprived of GRASP1 indeed showed impairment in the surface levels of AMPA-R subunits GluA1 and GluA2 and defects in LTP induction and spatial memory behavioral tests. In addition, glycine treatment, an LTP-like stimulus, was observed to potentiate the association between the three proteins in cultured neurons.

Two different GRASP1 mutations, found in patients affected by intellectual disability, were also investigated. The mutations, although having opposite effects on the levels of interaction between GRASP1 and GRIP1, produced the same downstream reduction in surface levels of AMPA-Rs, suggesting a tightly regulated mechanism (Chiu et al., 2017).

Another group found GRASP1 in the context of AMPA-R recycling and LTP (Lu et al., 2017). The authors identified GRASP1 as a key protein whose transcription is regulated by the translational regulator cytoplasmic polyadenylation element binding protein 2 (CPEB2). As expected, the absence of CPEB2 in a knockout mouse model led to reduction in the levels of GRASP1 and secondarily to a decrease in the surface level of AMPA-Rs, impaired LTP and defective performances in spatial and contextual fear memory tests.

### Hebbian Plasticity—LTD

LTD induction is usually followed by a rapid internalization of AMPA-Rs with a consequent rerouting of the receptors towards the lysosomal compartment for degradation, thus preventing their entrance in the recycling endosomes system (Lüscher and Malenka, 2012; Huganir and Nicoll, 2013).

One of the most important player in these events is PICK1 (Hanley, 2008). This protein, as introduced in the “Basal Constitutive Recycling” section, binds to the C-terminus of GluA2 AMPA-R subunit and promotes its internalization (Xia et al., 1999; Perez et al., 2001; Terashima et al., 2004); this interaction was shown to be necessary for the induction of NMDA-R-dependent LTD (Kim et al., 2001; Terashima et al., 2008). On the other hand, PICK1 has also been shown to promote the intracellular retention of GluA2 in recycling endosomes, thus allowing maintenance of depotentiation phenomena (Lin and Huganir, 2007; Madsen et al., 2012). On the other hand, PICK1 was also associated with increased surface delivery of CP-AMPA-Rs which could explain why its overexpression induces synaptic potentiation phenomena that occludes further LTP induction whereas its knockdown blocks LTP (Terashima et al., 2004, 2008; Clem et al., 2010). A detailed mechanism of the role of PICK1 in these processes is still lacking; the most likely hypothesis is that PICK1-mediated removal of GluA2 occurs both in LTP (only in the first phases) and LTD (permanently) with the receptor being substituted by CP-AMPA-Rs only in the first case with a mechanism that still needs to be elucidated.

Recently, Fiuza et al. (2017) further elucidated the mechanism through which PICK1 mediates GluA2 removal from the surface membrane. The authors showed that PICK1 is recruited to clathrin-coated pits by interacting with the adaptor protein complex AP-2, an association enhanced by LTD-like NMDA-R activation. In addition, PICK1 was also able to promote dynamin polymerization by interacting with its GTPase domain. This work interestingly suggests that PICK1 not only links AMPA-Rs to the endocytic machinery to enhance its internalization but that it is directly able to promote the activity of two crucial players in endocytosis events, AP-2 and dynamin (Fiuza et al., 2017).

Another protein, protein kinase C and casein kinase II substrate in neurons (PACSIN1), was recently found to regulate AMPA-R recycling that occurs after LTD-like phenomena (Widagdo et al., 2016). PACSIN1 was already known to regulate endocytosis of GluA2 after NMDA-R stimulation through interaction with PICK1 in a phosphorylation-dependent manner (Anggono et al., 2013). The authors here identified a novel specific role of PACSIN1 in the recycling step of AMPA-Rs that follows internalization after NMDA-Rs stimulation. This activity was also mediated by the interaction of PACSIN1, through specific serine residues, with PICK1, which once again shows a bidirectional role in being able to regulate both internalization and exocytosis.

The protein Cortactin, which was previously associated with actin dynamics, was recently identified to bind the AMPA-R subunit GluA2, regulating its fate in the endo-lysosomal system (Parkinson et al., 2018). Cortactin was found to prevent specifically GluA2/GluA3-containing AMPA-R from being directed towards the lysosomal compartment and subsequently degraded in basal conditions, possibly by retaining them in the early endosomes and thus favoring their redirection to recycling endosomes. As expected, this association appeared to decrease in the context of a chemical LTD protocol, where GluA2-containing receptors are rapidly degraded. On the other hand, cortactin knockdown impaired the induction of LTD, likely due to an occlusion mechanism caused by reduced levels of surface AMPA-Rs.

## Conclusion

Although AMPA-R recycling has been known for a long time now (Huganir and Nicoll, 2013), many details are still missing for a full picture to be obtained. The crucial role that the existence of this continuously cycling pool of receptors plays in the phenomena of synaptic plasticity, which are considered the biological correlates of the higher cognitive function of learning and memory, makes our in-depth understanding of this pathway extremely important. The importance of these processes is also inferable by the high number of recently published manuscripts that describe newly discovered adaptor proteins or new details of the exocytosis mechanism.

Thus, more efforts are needed to fully describe the mechanisms that produce the basal constitutive cycling of the receptor to then better characterize the molecular pathways that exploit this pool for all synaptic plasticity phenomena.

We would like to underline the importance of a comprehensive analysis of the AMPA-R subunits behavior when studying adaptor proteins, to further elucidate the specific mechanisms regulating different AMPA-R tetramers.

## Author Contributions

EM and MP conceived, wrote and revised the manuscript.

## Conflict of Interest Statement

The authors declare that the research was conducted in the absence of any commercial or financial relationships that could be construed as a potential conflict of interest.
